# Chronic impairment of neurovascular coupling and cognitive decline in young survivors of severe traumatic brain injury

**DOI:** 10.1007/s11357-025-01683-w

**Published:** 2025-05-14

**Authors:** Zsofia Dina Magyar-Sumegi, Mark Csendes, Dominika Lendvai-Emmert, Gabriella Sebestyen, Viktoria Tamas, Szabolcs Bandi, Andras Czigler, Andriy Yabluchanskiy, Stefano Tarantini, Zoltan Ungvari, Endre Czeiter, Krisztina Amrein, Gergely Orsi, Gabor Perlaki, Andras Buki, Peter Toth

**Affiliations:** 1 Department of Neurosurgery, Medical School, University of Pecs, Pecs, Hungary; 2 Department of Psychiatry and Psychotherapy, Medical School, University of Pecs, Pecs, Hungary; 3 Doctoral School of Clinical Neurosciences, Medical School, University of Pecs, Pecs, Hungary; 4 Vascular Cognitive Impairment, Neurodegeneration and Healthy Brain Aging Program, Department of Neurosurgery, University of Oklahoma Health Sciences Center, Oklahoma City, OK USA; 5 Doctoral College Health Sciences Division/Institute of Preventive Medicine and Public Health, International Training Program in Geroscience, Semmelweis University, Budapest, Hungary; 6 HUN-REN–PTE Clinical Neuroscience MR Research Group, University of Pecs, Pecs, Hungary; 7 Molecular Medicine Research Group, Szentagothai Research Centre, University of Pecs, Pecs, Hungary; 8 Department of Neurosurgery, Faculty of Medicine and Health, Orebro University, Orebro, Sweden

**Keywords:** Neurovascular hyperemia, Autoregulation, CBF, Brain injury, Cognitive impairment, IGF-1

## Abstract

Severe traumatic brain injury (TBI) leads to chronic cognitive decline, imposing a significant societal burden. The regulation of cerebral blood flow (CBF) is critical for cognitive function, and acute disruptions in CBF regulation predict poor TBI outcomes. However, the long-term effects of TBI on CBF regulation and their association with cognitive function remain poorly understood. This study aimed to investigate whether severe TBI results in chronic CBF dysregulation and whether this contributes to long-term cognitive deficits. Additionally, we examined the role of TBI-induced insulin-like growth factor 1 (IGF-1) deficiency in cerebrovascular dysfunction. We assessed cognitive function, basal CBF (via phase contrast MRI), CBF autoregulation (via transcranial Doppler), and neurovascular coupling (NVC) in 33 TBI survivors (mean age 37.6 years, ~ 10 years post-injury) and 21 age-matched healthy controls. Serum IGF-1 levels were also measured. TBI survivors exhibited significant impairments in memory and executive function compared to controls. While basal CBF and autoregulation remained intact, NVC responses were chronically impaired and correlated with cognitive deficits. However, IGF-1 levels did not differ between groups and were not associated with NVC impairment or cognitive function. Our findings indicate that severe TBI results in chronic impairment of neurovascular coupling, which likely contributes to long-term cognitive deficits. These results highlight the need for further research to identify underlying neurovascular mechanisms and develop interventions to restore NVC and cognitive function in TBI survivors.

## Introduction

Severe traumatic brain injury (sTBI) is a leading cause of long-term disability, with survivors often experiencing persistent cognitive decline that significantly impacts their quality of life and socioeconomic integration [1, 2]. While the immediate effects of TBI are well-documented, emerging evidence suggests that secondary injury mechanisms contribute to long-term neurological dysfunction, including deficits in memory, attention, executive function, and information processing speed [3, 4]. However, the precise pathophysiological processes underlying chronic cognitive impairment in TBI survivors remain incompletely understood.

Cerebral blood flow (CBF) regulation plays a crucial role in maintaining cognitive function. TBI was shown to profoundly affect regulatory mechanisms of CBF [5, 6], contributing significantly to secondary brain injury. Accordingly, after brain trauma autoregulation of CBF is impaired, which determines outcome of TBI [5–9] and likely contributes to cognitive dysfunction observed in these patients [5–9]. Neurovascular coupling, the mechanism adjusting local perfusion for an active brain region, is also impaired after TBI [10–12]. Preclinical and clinical studies demonstrate that in various pathophysiological conditions impaired neurovascular coupling contributes to the development of cognitive impairment [13–15]. It is further substantiated by our previous findings that selective impairment of neurovascular hyperemia is associated with decreased cognitive function in mice [16].

Previous studies have implicated disruptions in the growth hormone–insulin-like growth factor 1 (GH-IGF-1) axis as a key factor in post-TBI decline in brain health [17, 18]. IGF-1 is essential for cerebromicrovascular homeostasis, and its deficiency has been linked to impaired NVC [13, 19, 20] and CBF regulation [21]. Given that IGF-1 deficiency occurs in up to 66% of TBI survivors [22], it is plausible that chronic IGF-1 dysregulation may contribute to long-term cerebrovascular dysfunction and cognitive impairment in this population.

Building on prior research, we hypothesized that (1) severe TBI results in chronic impairments in NVC and CBF autoregulation, which are associated with cognitive deficits, and that (2) TBI-induced IGF-1 deficiency contributes to these cerebrovascular impairments. To test these hypotheses, we assessed neurovascular function and cognitive performance in young survivors of severe TBI, utilizing multimodal imaging and neuropsychological assessments. Our findings provide novel insights into the chronic impact of TBI on cerebrovascular regulation and its role in cognitive decline, with implications for future therapeutic strategies aimed at restoring neurovascular function in TBI survivors.

## Methods

### Participants

This study was approved by the National Ethics Committee (1828–3/2020/EUIG) in Hungary. Between 2021 and 2023, we have processed data from 471 traumatic brain injury patients, who had been previously treated at the Department of Neurosurgery, Medical School, University of Pecs, Hungary. We contacted the patients either by phone or via mail to invite them to participate in our follow-up study. Seventy-five patients visited our outpatient section for follow-up examination. We excluded patients with previous mild and moderate brain injury (GCS ≥ 9), as well as people over 50 years of age. Finally, we studied 33 patients with severe traumatic brain injury in the past (87.88% male, mean age 37.61, average time after trauma 9.98 years, average GCS 5.39). They were injured in a road accident (87.88%) and falls (9.09%). Suicide attempts also occurred (3.03%). In addition to the TBI group, age- and gender-matched healthy control subjects were included in the present study (*n* = 21, 80.95% male, mean age 35.09). A detailed description of the participants involved in the present study is given in Table 1.

**Table 1:** Characteristics of the patients with traumatic brain injury and control subjects

	TBI patients *n* = 33		Control subjects *n* = 21	
Variables				
Sex	*n*	%	*n*	%
Male	29	87.88	17	80.95
Female	4	12.12	4	19.05
	Md	Mean (SD)	Md	Mean (SD)
Age at follow-up examination	40	37.61 (8.53)	34	35.09 (9.34)
Education, y	12	12.85 (2.73)	16	16.36 (1.23)
Time since injury, y	11	9.98 (6.90)	-	-
GCS	6	5.39 (1.66)	-	-
Pathology	*n*	%		
Contusion	14	42.42%	-	-
Epidural hemorrhage	3	9.09%	-	-
Subdural hemorrhage	15	45.45%	-	-
Subarachnoideal hemorrhage	12	36.36%	-	-
Cerebral edema	9	27.27%	-	-
Fracture	12	36.36%	-	-
Diffuse axonal injury	4	12.12%	-	-
Atrophy	1	3.03%	-	-
Comorbidity	*n*	%		
Epilepsy	1	3.03%	-	-
Hypertension	3	9.09%	-	-
Hypopituitarism	2	6.06%	-	-
Psychiatric	2	6.06%	-	-

Recruited patients and control participants visited our Department for a 1-day examination, during which blood samples were taken and a battery of neuropsychological tests, MRI flow measurements, and transcranial Doppler flowmetry were carried out. The tests took an average of 6–7 h per participant.

### Neuropsychological testing

To map neurocognition in details, we assessed executive function, memory, attention, and language skills. The following tests were used: Rey Auditory Verbal Learning Test, Rey–Osterrieth Complex Figure Test, Digit Span Forward, Digit Span Backward, Frontal Assessment Battery, Trail Making Test (parts A and B), Controlled Oral Word Association Test, Five Points Test, Toulouse-Pieron Test, Clock Drawing Test, Picture Naming Test of Dean Woodcock Neuropsychological Battery and Test of Nonverbal Intelligence [23]. Before starting to assess neurocognitive function, we assessed the outcome of TBI by determining Extended Glasgow Outcome Scale [24]. For statistical analysis, we created aggregated variables for certain tests. In the case of the Rey Auditory Verbal Learning Test, we added the values of three variables, which include immediate recall, learning, and the results of forgetting [25]. In the case of the Rey–Osterrieth Complex Figure Test, three variables were also combined, which show the results of copying and immediate and delayed recall. Results of the fluency tests (Controlled Oral Word Association Test, Five Points Test) were also combined.

The test series was usually completed in one sitting, taking an average of 2 h in the early morning of a given day.

### Detailed description of neuropsychological tests

1. *Frontal Assessment Battery:* The test is mainly used to detect frontal lobe dysfunction, takes about 10 min to complete, and consists of six subtests. It measures conceptualization and abstract thinking (similarities), mental flexibility (verbal fluency), motor programming (Luria’s three-step test), resistance to interference (conflict instruction), inhibitory control (Go/No-go task), and environmental autonomy. A maximum of 18 points can be achieved.
2. *Test of Nonverbal Intelligence, Third Edition (TONI-3):* A test of nonverbal intelligence (spatial-visual and numerical abilities), with an administration time of approximately 20 min. It consists of 45 tasks, each with several alternatives to choose from in order to match the missing part of the mosaic.
3. *Controlled Oral Word Association Test*: In addition to assessing executive functions, it evaluates linguistic functions and semantic memory. In the phonological part, participants must generate words within 1 min using the given initial letters (F, A, S). Subjects must avoid repetition of words, grammatical variations of the same words, and geographical and personal names. In the semantic/categorization part, participants must recall words from different categories (animals, vegetables, vehicles) within 1 min. The test takes approximately 7–8 min. For the phonological part, the average number of words generated is about 30–40, while for the categorization part, it is about 50–60 words.
4. *Trail Making Test (Part A and B):* Both parts of the test can be completed in a few minutes and measure visuomotor and psychomotor speed, focused attention, flexible thinking, working memory, visuomotor coordination, visuospatial ability, and visual concept formation (frontal lobe functions). In Part A, the patient must connect numbers in ascending order as quickly as possible (maximum of 100 s). In Part B, the patient must alternate between connecting numbers and letters in ascending order as quickly as possible, so that each number is followed by a letter (maximum of 300 s). On average, Part A can be completed in 20–30 s, and Part B in 60–70 s.
5. *Rey Auditory Verbal Learning Test (Version 3):* This test measures episodic long-term memory, learning ability, and verbal working memory. The test involves reading a 15-word list (List A) to the patient over 5 trials, with the patient recalling as many words as they can remember after each reading. After the fifth trial, an interfering task is administered, where the examiner reads a new 15-word list (List B) only once, and the patient must recall as many words as they can remember. This is followed by an immediate recall test of the words from List A and a delayed recall test of the words from List A after 20 min. The entire test takes about 8–10 min.
6. *Rey-Osterrieth Complex Figure Test:* This test measures visual organization and visual incidental memory. The first part of the task involves copying a figure, while the second part involves recalling and reconstructing the figure from memory after a certain amount of time. During both the copying and recall phases, the color tools used must be interchanged to match the order in which the figure is constructed. There is no time limit for copying or reproduction from memory, but the elapsed time should be recorded. The method allows for both quantitative and qualitative assessment of the subject’s performance. The figure is divided into 18 units and copying and recall performance are scored according to specific criteria. Through qualitative analysis of individual task solutions, visual perception, attentional processes, incidental visual and visuospatial memory, and various problems in organization and construction can be assessed. The test takes about 10 min.
7. *Toulouse-Pieron Attention Test:* It is a test of attention, concentration, and executive functions. The task involves finding predetermined shapes within 5 min. Scoring is done by subtracting the number of errors from the total number of items reviewed and dividing this by the total number of items reviewed to obtain the individual’s score (expressed as a percentage).
8. *Digit Span Forward and Backward:*

*Forward:* In this exercise, the participants must recall digits in the same order. There are seven sequences of digits, starting with 3 digits and increasing by one digit per sequence, up to 9 digits in the last sequence. The performance is measured by the number of sequences the individual can recall correctly (with each sequence offering two different possibilities). This test measures verbal auditory attention capacity and short-term memory.

*Backward:* In this task, numbers are recalled in reverse order. There are seven sequences, starting with 2 digits and increasing by one digit per sequence, up to 8 digits in the last sequence. Performance is based on the number of sequences the individual can recall correctly without error (with each sequence offering two different possibilities). This test primarily measures central executive functioning and verbal working memory capacity.

9. *Pattern Fluency Test (Five-Point Test):* This is a non-verbal fluency test and one of the subtests in the area of attentional/executive functions. Five points are pre-set in each of 35 cells, and the task is to connect them to form as many unique patterns as possible. The number of unique, non-repeated patterns indicates the person’s performance. The test takes approximately three minutes to complete.
10. *Clock-Drawing Test:* It is a reliable screening tool for cognitive impairment, particularly dementia, as it evaluates multiple cognitive domains, including executive function, attention, language skills, frontal lobe function, and visuospatial skills. To successfully complete the clock drawing, an individual must understand verbal instructions, encode them into short-term memory, and use visual construction skills to draw the clock. The outline of the clock and the numbers are drawn by the participant, who then has to place the hands according to the given instructions.

### Magnetic resonance imaging (MRI) – cerebral blood flow analysis

Out of the 33 TBI patients included in the study, MRI scans were performed on 30 patients (86.67% male, mean age 37.50 years, mean post-trauma time 9.72 years, mean initial GCS 5.39). Three patients could not be scanned due to untreated epilepsy, a shunt, or claustrophobia. Additionally, 3 control volunteers were excluded due to claustrophobia, resulting in a total of 18 control volunteers with MRI scans (83.33% male, mean age 34.50).

MRI measurements were performed on a 3 T MRI scanner (MAGNETOM Prisma^Fit^, Siemens Healthcare, Erlangen, Germany) with a 20-channel Head/Neck coil. The flow was measured in the M1 segments of both left and right middle cerebral arteries (MCAs) using a parasagittal two-dimensional single-slice phase-contrast (PC) sequence with peripheral pulse gating and the following parameters: TR/TE = 89.22/9.03 ms; flip angle = 15 degrees; slice thickness = 4 mm; FOV = 140 × 140 mm^2^; matrix size = 256 × 256 interpolated to 512 × 512; receiver bandwidth = 130 Hz/pixel; averages = 3; number of phases = 25; velocity encoding (VENC) = 100 cm/s in through-plane direction. The imaging plane was arranged perpendicular to the longitudinal axis of the vessels using a native 3D time-of-flight MR angiography (TOF-MRA) with the following parameters: TR/TE = 22/3.86 ms; flip angle = 18 degrees; slice thickness = 0.7 mm; FOV = 167 × 222 mm^2^; matrix size = 202 × 384; receiver bandwidth = 178 Hz/pixel; 4 overlapping (27.08%) slabs; a total of 153 axial slices (48 slices/slab). Flow analysis of PC MRI data were performed using Argus software (Leonardo workstation; Siemens Healthcare, Erlangen, Germany). After loading the magnitude, phase, and rephased images into Argus, vessel contour of MCA was manually outlined on the first cardiac phase image and then automatically propagated to all other cardiac phases. The propagated contours (i.e., automatically outlined contours) were carefully checked for each cardiac phase and manually adjusted to ensure accurate vessel boundary delineation. To avoid phase aliasing near vessel borders, velocity range was adjusted from ± 100 to − 50/+ 150 cm/s. After applying background correction, average flow (ml/s) and average MCA area (cm^2^) were automatically calculated by the software.

### Measurement of neurovascular coupling and autoregulation of cerebral blood flow

Chronic changes of the regulatory mechanisms of cerebral blood flow were assessed by functional transcranial Doppler (fTCD) in 23 patients (86.96% male, mean age 37.04, average time after trauma 10.09 years, average GCS 5.48) and 18 controls (77.78% male, mean age 35.06). Flow rate in middle cerebral arteries was examined, up to a depth of 45–60 mm. During the surveys, the systolic, diastolic, and average flow velocity values, as well as the pulsation index, were recorded, using a continuous finger cuff blood pressure measurement device. After recording resting hemodynamic parameters, we examined the patient’s cerebral blood flow responses during verbal fluency test and the Trail Making Test (parts A, B). To determine neurovascular coupling, we assessed the % changes of cerebrovascular conductance index (CVCi%) calculated as the ratio of cerebral blood flow (CBF) and mean arterial pressure (MAP) [26]. The CVCi results measured on the left and right side were averaged during each neurocognitive test. In addition to the neurovascular coupling, we determined autoregulatory function by obtaining systolic flow index (Sxa) and average flow index (Mxa). In brief, both Sxa and Mxa were calculated as moving linear correlation coefficients between cerebral blood flow velocity measured in the middle cerebral artery and arterial blood pressure. Specifically, Sxa was computed using systolic values of flow velocity and blood pressure, while Mxa utilized mean values. The calculations were performed using data from a 300-s window, with values averaged over consecutive 10-s intervals [27].

### Serum sample collection and IGF-1 and IGFBP-3 measurements

Following venipuncture, whole blood was collected into serum separator tubes and allowed to clot for 45 ± 15 min at room temperature. The samples were then centrifuged at 1500 × *g* for 10 min. The resulting serum supernatant was aliquoted into 0.5 mL portions in cryovials and stored at − 80 °C until analysis. Measurements of insulin-like growth factor 1 (IGF-1) and IGF-binding protein 3 (IGFBP-3) were conducted at the Department of Laboratory Medicine, University of Pécs. Most circulating IGF1 is bound to IGFBP3 (about 80%), forming a complex with the acid-labile subunit (ALS), which protects IGF1 from proteolytic degradation [28]. Quantification was performed using the Siemens IMMULITE 2000 platform (Siemens Healthineers AG, Forchheim, Germany) employing solid-phase, enzyme-labeled chemiluminescent immunometric assay (CLIA) technology with dedicated kits for IGF-1 (Cat# L2 KGF2) and IGFBP-3 (Cat# L2 KGB2).

### Statistical analysis

Statistical analyses were performed using IBM SPSS for Windows 23.0 (IBM Corp. Released 2015. IBM SPSS Statistics for Windows, Version 23.0. Armonk, NY: IBM Corp.) and GraphPad Prism 9 (GraphPad Software LLC., San Diego, CA, USA). Following descriptive statistics, the distribution of variables was checked in each case using Kolmogorov–Smirnov and Shapiro–Wilk tests. Depending on this, Pearson and Spearman correlations were used for relationship analysis, and *T*-tests/Mann–Whitney *U* analyses were used for difference analyses. In all cases, effect sizes were calculated for the analysis of differences. A *p* value less than 0.05 was considered statistically significant. Data are presented as mean (± 95% CI) for normally distributed variables and median (± 95% CI) for non-normally distributed variables.

## Results

### Severe traumatic brain injury leads to chronic decline in visual and verbal and working memory as well as executive function in young patients

**Fig. 1:**
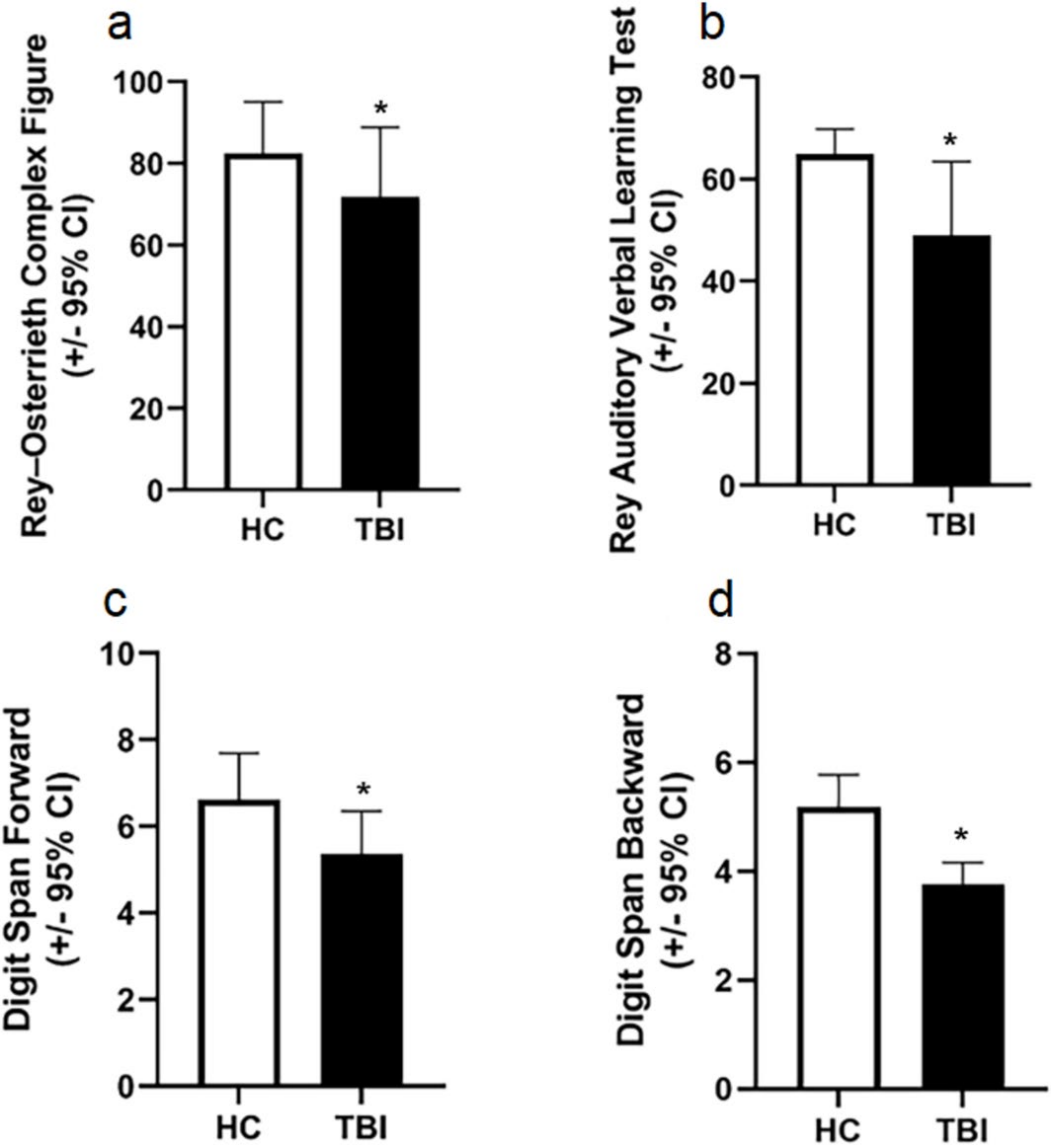
Severe traumatic brain injury leads to chronic decline in visual, verbal and working memory in young patients

**Fig. 2:**
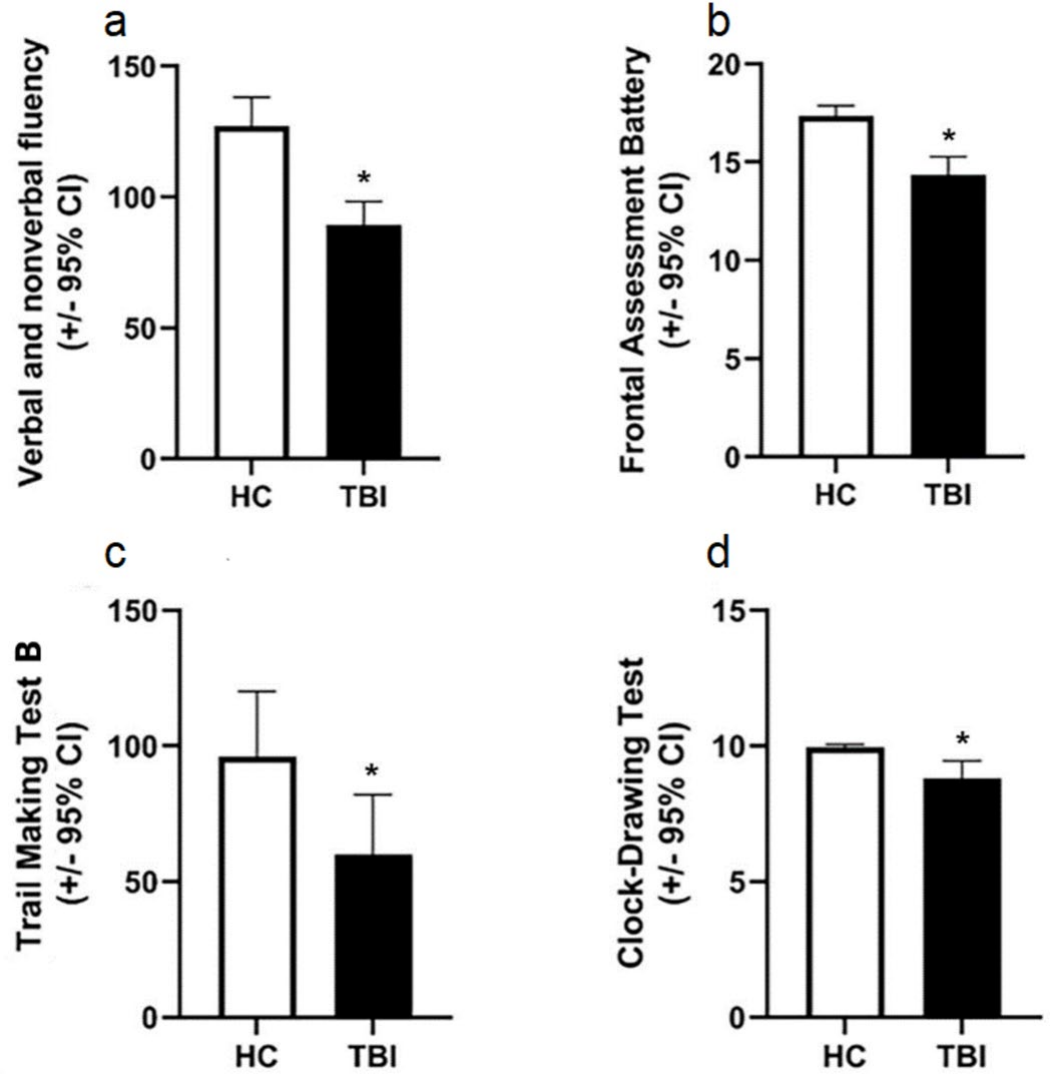
Severe traumatic brain injury leads to chronic decline in executive function (working memory, cognitive flexibility, and inhibitory control) in young patients

Young patients (*n* = 33, mean age 37.61) in the chronic period (average time after trauma 9.98 years) after severe traumatic brain injury (average GCS 5.39) exhibited a significantly impaired visual memory (*p* = 0.017), auditory verbal memory (*p* < *0.001*), short-term memory (*p* < *0.001*), working memory (*p* < *0.001*), and executive function (*p* < *0.001*), (*p* < *0.001*), (*p* < *0.001*), (*p* < *0.001*) compared to age-matched healthy young control volunteers (*n* = 21, mean age 35.09) (Figs. 1–2).

### Severe traumatic brain injury leads to chronic impairment of neurovascular coupling in young patients, which is not affected by IGF-1 level of patients

**Fig. 3:**
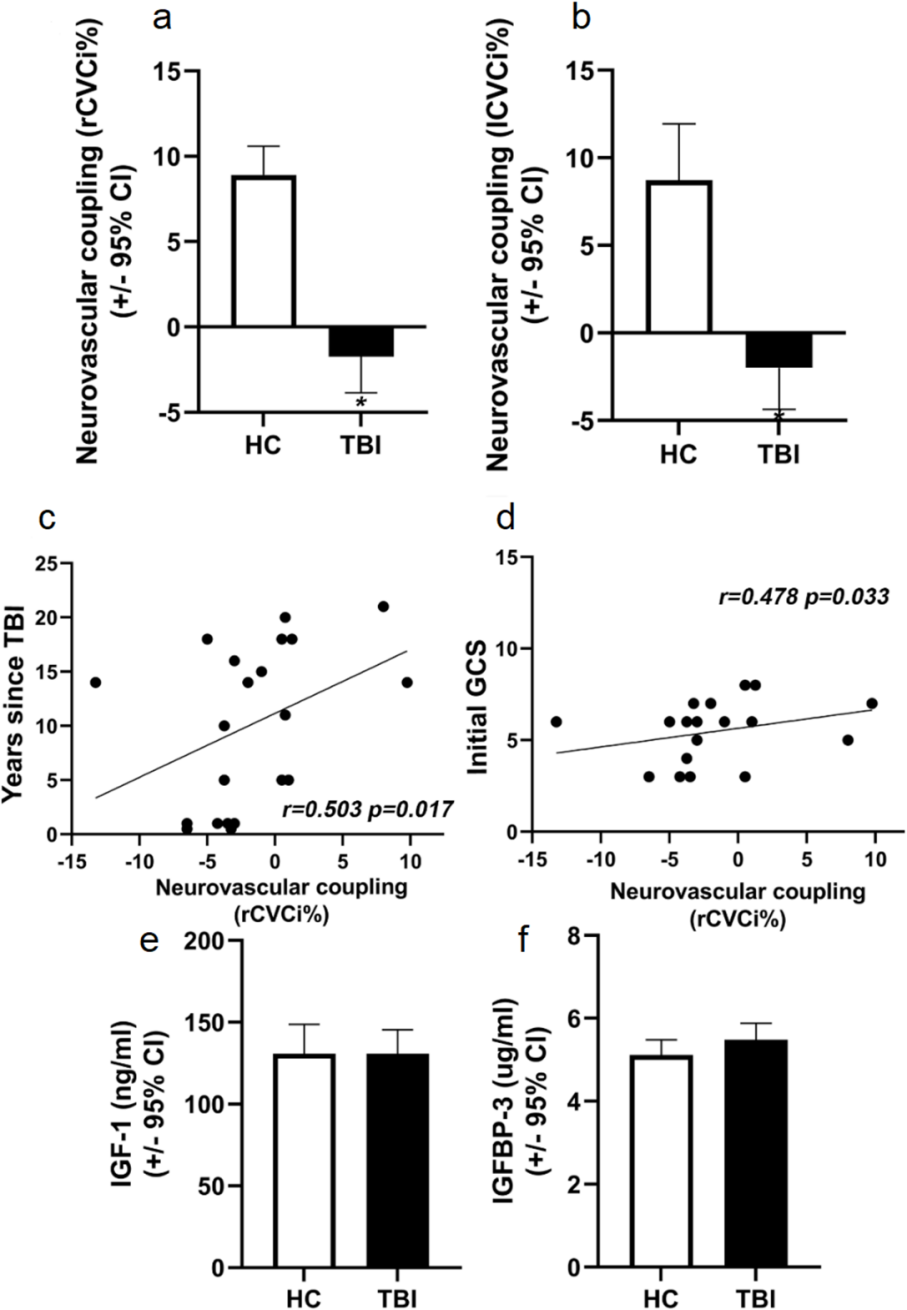
Severe traumatic brain injury leads to chronic impairment of neurovascular coupling in young patients

Young patients (*n* = 23, mean age 37.04) in the chronic period (average time after trauma 10.09 years) after severe traumatic brain injury (TBI) (average GCS 5.48) exhibited significantly (*p* < *0.001*) impaired neurovascular coupling responses measured in both middle cerebral arteries (MCA) as changes in cerebrovascular resistance (CVC) during neurocognitive tests compared to age-matched healthy young control volunteers (*n* = 18, mean age 35.06). It is important to note that neurovascular coupling responses of TBI patients significantly correlated positively with years since the traumatic event (*r*_*s*_ = 0.503; *p* = 0.017) and with the initial Glasgow Coma Scale (GCS) score (*r*_*s*_ = 0.478; *p* = 0.033). Importantly, we did not find differences in IGF-1 and IGF-1 binding protein levels between patients after TBI and age-matched healthy controls *t*(50) = 0.001; *p* = 0.999; *t*(50) = 1.317; *p* = 0.194 (Fig. 3).

### Impaired neurovascular coupling is associated with decline in cognitive function

**Fig. 4:**
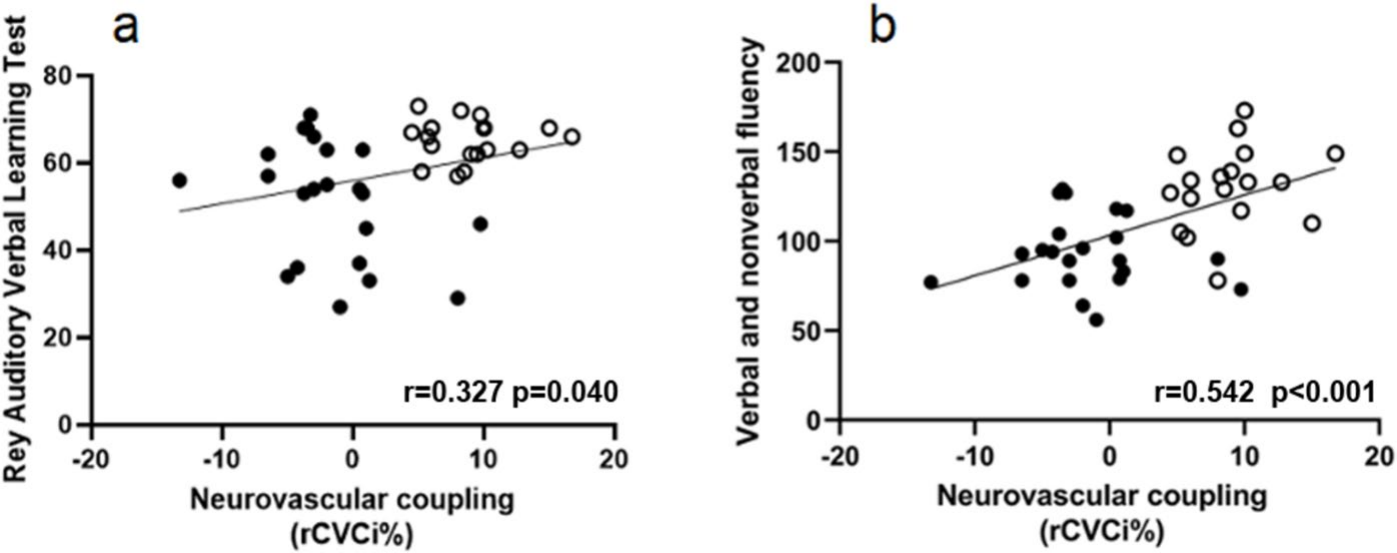
Impaired neurovascular coupling is associated with decline in cognitive function

Importantly, neurovascular coupling responses (as changes in cerebrovascular resistance during neurocognitive tests) significantly correlated with results on neuropsychological tests representing memory (*r*_*s*_ = 0.327; *p* = 0.040) and executive function (*r* = 0.542; *p* < *0.001*) in the studied groups of patients (TBI: *n* = 22, mean age 36.59, average time after trauma 10.14 years, average GCS 5.45; HC: *n* = 18, mean age 35.06). It has to be noted that statistical significance was not reached in the groups separately tested, most likely because of small number of patients studied (Fig. 4).

### Basal cerebral blood flow (CBF) and autoregulation of CBF are not affected in young survivors of severe traumatic brain injury

**Fig. 5:**
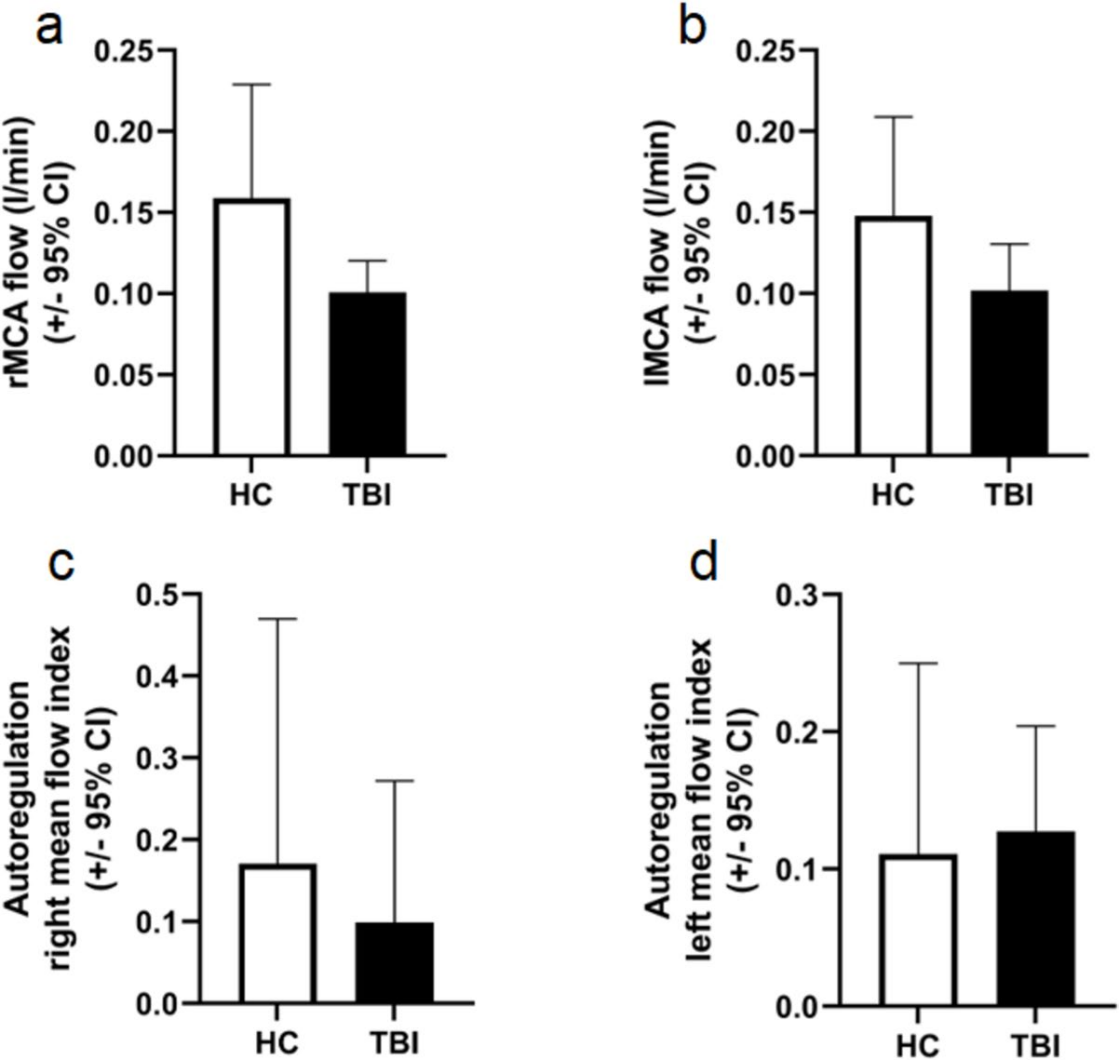
Basal cerebral blood flow (CBF) and autoregulation of CBF are not affected in young survivors of severe traumatic brain injury

Blood flow (l/min) measured by phase-contrast MRI in both middle cerebral arteries in the chronic period (average time after trauma 9.00 years) after severe traumatic brain injury (TBI) (average GCS 6.17) in young patients (*n* = 7, mean age 35.14) and in age-matched healthy young control volunteers (*n* = 4, mean age 25.75) was not different significantly (*p* = 0.072 and 0.057 on the right and left side, respectively). Autoregulation of cerebral blood flow (assessed as the moving correlation coefficient between mean cerebral blood flow velocity and mean arterial blood pressure) showed no differences (*p* = 0.372 and 0.829 on the right and left side, respectively) (Fig. 5).

## Discussion

Severe TBI has a profound impact across all ages, races, and socioeconomic classes in societies worldwide, owing to its high mortality rate and the significant disabilities experienced by survivors. While the primary injury results directly from the impact, the subsequent secondary brain injury, triggered by the initial insult, significantly influences patient outcomes and recovery [3, 4, 29]. It is now understood that the consequences of TBI are not limited to a few months or even a year; rather, chronic effects can emerge and evolve long after the initial injury, with deterioration observed many years post-trauma [30]. Therefore, TBI should be considered a chronic condition initiated by the injury, affecting neurological function even decades later [30–32]. This perspective is supported by previous studies demonstrating a direct relationship between the number and severity of TBIs and the extent of cognitive deficits [33, 34]. Our current findings further substantiate this, revealing that visual, verbal, and working memory, as well as executive function, remain impaired years after severe TBI when compared to healthy controls. Interestingly, we found a negative correlation between the time since trauma and auditory verbal memory (data not shown), suggesting that TBI results in different dynamics of impairment of different cognitive domains likely due to different TBI-related pathomechanisms involved. It would be important to assess in a prospective study how cognitive function improves in this patient population, and how it relates to these individuals’ reintegration to the society with special focus on the changes of the affected domains.

In this study, we provide evidence that young survivors of severe TBI exhibit chronic impairment of neurovascular coupling (NVC), which correlates with cognitive deficits. This suggests that the neurovascular unit does not fully recover from the injury, potentially leading to chronic mismatches between neuronal metabolic demand and blood supply. Previous studies have demonstrated that acute dysregulation of cerebral blood flow (CBF) autoregulation strongly predicts poor outcomes in TBI [5–9]. However, our results indicate that while basal CBF and autoregulation normalize over time, NVC remains persistently impaired. This persistent dysregulation may contribute to the long-term cognitive deficits observed in TBI survivors, as impaired functional hyperemia can reduce nutrient and oxygen delivery to active brain regions [16, 35, 36]. When interpreting the relationship between cognitive performance and neurovascular coupling, factors like hemispheric dominance, handedness, and language lateralization have to be considered. Although 97.56% of participants in our study were right-handed [37] suggesting that most of them had left-hemisphere language dominance, the right hemisphere is crucial for cognitive functions like visuospatial working memory and attentional control, relevant to tasks assessing neurovascular coupling, such as the Trail Making Test [38, 39]. It also has to be noted that following traumatic brain injury, both neural activation and synaptic activity are often diminished, particularly in the affected brain regions. This reduction may be transient or long-lasting, depending on the severity and extent of the injury, as well as the efficacy of subsequent recovery processes. Long-term synaptic dysfunction may also develop, which can persist for years after the initial trauma and contribute to sustained impairments in cognitive function. Therefore, our observed perfusion disturbances interpreted as NVC impairment could be a result of neuronal injury and diminished spontaneous neural activity in higher-order cognitive regions [40].

Interestingly, we found that NVC impairment correlated with the time elapsed since injury, suggesting a dynamic recovery process. However, the correlation between NVC and cognitive function supports the notion that these vascular changes are functionally significant and not merely epiphenomena of brain injury. Given that chronic neurovascular dysfunction is also implicated in neurodegenerative diseases such as Alzheimer’s disease and vascular contributions to cognitive impairment and dementia (VCID) [14, 41–43], our findings raise the possibility that TBI survivors may be at increased risk for long-term neurodegeneration and dementia due to persistent vascular dysfunction.

Mechanisms of TBI-induced chronic impairment of NVC are likely multifaceted. Previous studies have implicated IGF-1 deficiency as a key contributor to cerebrovascular dysfunction and cognitive impairment [19]. Given that TBI-induced hypopituitarism is relatively common (affecting up to 66% of TBI survivors [22]), we hypothesized that IGF-1 deficiency might underlie the observed NVC impairments. However, we found no significant difference in IGF-1 levels between TBI survivors and healthy controls. While this finding does not rule out a role for IGF-1 in acute TBI-related cerebrovascular dysfunction, it suggests that other mechanisms—such as chronic inflammation, oxidative stress, mitochondrial dysfunction, or blood–brain barrier (BBB) disruption—may drive long-term NVC impairment [10, 44, 45]. Future studies should explore these alternative pathways to identify therapeutic targets for improving cerebrovascular function in TBI survivors, as well as the possible role of other IGF-1 binding proteins.

The findings of our study have significant clinical and research implications, particularly in understanding and addressing the long-term consequences of severe TBI. The strong association between chronic NVC impairment and cognitive decline underscores the urgent need for targeted therapeutic interventions aimed at restoring functional hyperemia. Approaches such as vascular-targeted rehabilitation programs, pharmacological strategies [15]—including antioxidant protocols [46]—and neuromodulation techniques [47] may hold promise in mitigating the effects of NVC dysfunction and improving cognitive outcomes in TBI survivors. Beyond potential interventions, our study highlights the critical need for longitudinal investigations. Given that our data provide only a cross-sectional snapshot of chronic NVC impairment, future research should track the trajectory of neurovascular and autoregulatory dysfunction and cognitive performance over time. It remains unclear whether these impairments progressively worsen, stabilize, or exhibit signs of spontaneous recovery. Understanding these patterns could help refine treatment strategies and identify optimal time windows for therapeutic intervention. Moreover, our results contribute to the growing body of evidence linking TBI to an increased risk of neurodegenerative diseases [48]. The persistent vascular dysfunction observed in TBI survivors bears striking similarities to VCID [14]. This raises the possibility that chronic NVC impairment may predispose TBI survivors to an accelerated cognitive decline later in life. Future studies should explore whether vascular-targeted interventions not only improve cognitive function in TBI survivors but also reduce their long-term susceptibility to neurodegenerative conditions.

In conclusion, this study provides compelling evidence that NVC remains chronically impaired for years in young survivors of severe TBI, representing a key mechanism underlying long-term cognitive deterioration. While basal CBF and autoregulation appear to recover over time, the persistent dysfunction in NVC responses suggests a lasting disruption in the ability of the brain to regulate perfusion in response to cognitive demands. Given the well-established link between cerebrovascular health and cognitive function, these findings emphasize the urgent need for further research into the vascular pathophysiology driving this dysfunction. A deeper understanding of the mechanisms underlying chronic neurovascular impairment may pave the way for targeted therapeutic strategies aimed at restoring neurovascular integrity and mitigating post-traumatic cognitive decline. Future studies should focus on identifying modifiable risk factors and exploring interventions—whether pharmacological, rehabilitative, or neuromodulatory—to improve neurovascular function and enhance long-term outcomes in TBI survivors.

## Data Availability

The data that support the results of this study are available from the corresponding author upon reasonable request.
